# Deciphering Cell-Type-Specific Gene Expression Signatures of Cardiac Diseases Through Reconstruction of Bulk Transcriptomes

**DOI:** 10.3389/fcell.2022.792774

**Published:** 2022-02-18

**Authors:** Xiaobin Wu, Xingyu Zhao, Yufei Xiong, Ming Zheng, Chao Zhong, Yuan Zhou

**Affiliations:** ^1^ Department of Biomedical Informatics, Center for Noncoding RNA Medicine, School of Basic Medical Sciences, Peking University, Beijing, China; ^2^ MOE Key Laboratory of Molecular Cardiovascular Sciences, Peking University, Beijing, China; ^3^ Beijing Key Laboratory of Tumor Systems Biology, Department of Immunology, School of Basic Medical Sciences, Institute of Systems Biomedicine, Peking University, Beijing, China; ^4^ Department of Physiology and Pathophysiology, School of Basic Medical Sciences, Peking University, Beijing, China

**Keywords:** cardiac diseases, transcriptome reconstruction, single-cell RNA sequencing, cellular composition, monocyte, fibroblast

## Abstract

Cardiac diseases compose a fatal disease category worldwide. Over the past decade, high-throughput transcriptome sequencing of bulk heart tissues has widened our understanding of the onset and progression of cardiac diseases. The recent rise of single-cell RNA sequencing (scRNA-seq) technology further enables deep explorations of their molecular mechanisms in a cell-type-specific manner. However, due to technical difficulties in performing scRNA-seq on heart tissues, there are still few scRNA-seq studies on cardiac diseases. In this study, we demonstrate that an effective alternative could be cell-type-specific computational reconstruction of bulk transcriptomes. An integrative bulk transcriptome dataset covering 110 samples from 12 studies was first constructed by re-analysis of raw sequencing data derived from the heart tissues of four common cardiac disease mouse models (myocardial infarction, dilated cardiomyopathy, hypertrophic cardiomyopathy, and arrhythmogenic right ventricular cardiomyopathy). Based on the single-cell reference covering four major cardiac component cell types and 22 immune cell subtypes, for each sample, the bulk transcriptome was reconstructed into cellular compositions and cell-type-specific expression profiles by CIBERSORTx. Variations in the estimated cell composition revealed elevated abundances of fibroblast and monocyte during myocardial infarction, which were further verified by our flow cytometry experiment. Moreover, through cell-type-specific differential gene expression and pathway enrichment analysis, we observed a series of signaling pathways that mapped to specific cell type in diseases, like MAPK and EGFR1 signaling pathways in fibroblasts in myocardial infarction. We also found an increased expression of several secretory proteins in monocytes which may serve as regulatory factors in cardiac fibrosis. Finally, a ligand–receptor analysis identified key cell types which may serve as hubs in cellular communication in cardiac diseases. Our results provide novel clues for the cell-type-specific signatures of cardiac diseases that would promote better understanding of their pathophysiological mechanisms.

## Introduction

The onset and progression of complex cardiac diseases often involve a variety of genes and features through systemic gene expression alterations. With the popularity of sequencing technologies, RNA sequencing of bulk tissues (bulk RNA-seq) has generated a huge amount of data about transcriptomic alterations in cardiac diseases in the last decade (Wang et al., 2009). Such bulk RNA-seq data sketch the overall transcriptomic landscape of cardiac disease at the whole tissue level, which has provided useful clues for investigating cardiac disease genes and pathways. Nevertheless, according to the recently published comprehensive cell atlas of healthy hearts in human and mouse, the heart tissue is typically composed of a variety of cell types, including but not limited to cardiomyocyte, endothelial cell, fibroblast, and smooth muscle cell (Litviňuková et al., 2020; Tabula Muris, 2020). Besides this, it has also been observed that multiple groups of immune cells would be recruited or activated during some cardiac diseases like heart failure (Martini et al., 2019). Therefore, in order to comprehensively understand the molecular mechanism of cardiac diseases, it is necessary to dissect the bulk transcriptome to the single cell or, at least, to the single cell type level.

The recently emerged single-cell RNA sequencing (scRNA-seq) technology should be an outstanding approach to explore transcriptome dynamics at the resolution of a single cell (Shapiro et al., 2013). Through scRNA-seq, many disease-associated mechanisms could be ascribed to specific cell types, and cell-type-specific therapeutic targets of cardiac diseases would be screened thereby—for example, Li et al. (2019) have defined endothelial heterogeneity in the healthy and injured heart through characterization of the gene expression signature in endothelial cells by scRNA-seq, and Farbehi et al. (2019) performed scRNA-seq to investigate the total non-cardiomyocyte fraction and fibroblast lineage cells from murine heart after a myocardial infarction surgery. However, most of these studies focused on one specific disease and only a part of cell types, where several intrinsic characteristics of popular scRNA-seq techniques like 10x Genomics have limited their application in cardiac disease research. First, the cost per sample of scRNA-seq is much higher than bulk RNA-seq; therefore, the scRNA-seq data are often of a small sample size (some even have no biological replicate) (Farbehi et al., 2019; Li et al., 2019; Martini et al., 2019) and thus exhibit lack of consideration of individual variations. Second, the single-cell suspension preparation procedure in the scRNA-seq pipeline is very challenging to cell survival, and it is known that some important cell types, like cardiomyocytes and neurons, are fragile to such procedure, especially in disease conditions when the cell vitality is already weak (Tucker et al., 2020; Zhou et al., 2020). Finally, the coverage of gene expression quantification in scRNA-seq data (usually covering 10^3^ genes) is also substantially compromised in comparison with bulk RNA-seq data (usually covering 10^4^ genes), which limited its usage for depicting the whole-transcriptome-level alterations during the disease processes (Hou et al., 2020). Given the aforementioned intrinsic limitation of scRNA-seq technology, an alternative approach is to dissect bulk transcriptomes to specific cell types. Indeed several methods have been established to estimate cell type compositions from the bulk transcriptomes of tissue samples, such as MuSiC (Wang et al., 2019), Bisque (Jew et al., 2020), and SCDC (Dong et al., 2021), but only very recently, the CIBERSORTx (Newman et al., 2019) method has been enabled to not only enumerate cell type abundance but also infer cell-type-specific gene expression profiles by integrating bulk RNA-seq series and a scRNA-seq reference. CIBERSORTx has shown promising results in cancer tissue studies where they have imputed tumor-infiltrating immune cell fraction and immune-cell-specific expression—for example, Li et al. (2020) identified 755 differentially expressed genes in CD4-GZMA T cell and further found a series of pathways related to the tumor microenvironment and immune response. In addition, Xu et al. (2021) have used CIBERSORTx to impute multiple immune-cell-specific expressions and systematically compared the expression difference of key genes in different cell types between tumor and control samples. We hereby employ CIBERSORTx as the proxy to deconvolute the rich resource of bulk transcriptomes of cardiac diseases into cell-type-specific gene expression profiles. More specifically, heart tissue RNA-seq data across various mouse models of myocardial infarction (MI), dilated cardiomyopathy (DCM), hypertrophic cardiomyopathy (HCM), and arrhythmogenic right ventricular cardiomyopathy (ARVC) were included as the input transcriptomes and the comprehensive single cell atlas of four non-immune heart cell types (cardiomyocyte, endothelial cell, fibroblast, and smooth muscle cell) (Tabula Muris, 2018), and 22 immune cell subtypes (LM22) (Newman et al., 2015) were considered as the reference. By this procedure, for each sample of bulk transcriptome, CIBERSORTx could provide the relative abundance of these 26 cell types and the specific expression profile of each cell type. We used these reconstructed expression data to explore the cell composition dynamics and cell-type-specific pathways involved in the four cardiac diseases. Moreover, flow cytometry was used to verify the number of monocytes, macrophages, and fibroblast from healthy state to MI state. Through the cell-type-specific biological analysis, we found some novel pathways related to specific diseases, like activated Wnt signaling pathway in DCM and EGFR1 signaling pathway in MI, and both of them could be ascribed to fibroblasts. Several increased secretory protein genes in monocytes in MI were identified to interact with the pathway-related genes in fibroblasts, which suggest their roles in cardiac fibrosis.

## Materials and Methods

### Bulk RNA-Seq Input Data Collection, Re-Quantification and Correction

**FIGURE 1:**
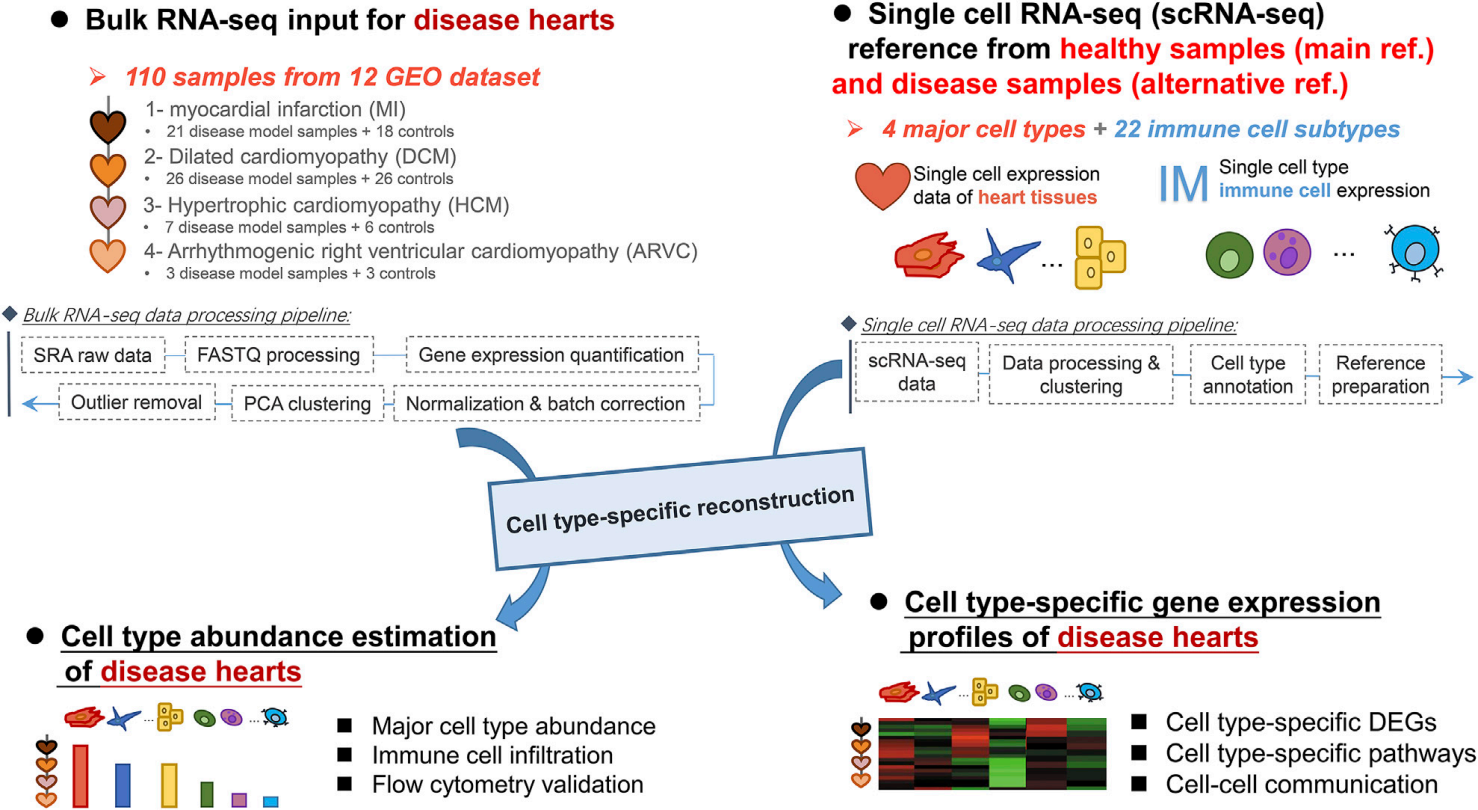
Workflow of the data processing and analysis pipeline.

The workflow of data processing and analysis is depicted in Figure 1. We first retrieved the bulk RNA-seq data of the heart tissue of a cardiac disease mouse model from NCBI GEO database (http://ncbi.nlm.nih.gov/geo/). By using the keyword combination [cardiac disease (All Fields) OR heart disease (All Fields)] AND [“Mus musculus” (Organism) AND “Expression profiling by high throughput sequencing” (Filter)], we have collected 17 bulk RNA-seq datasets that contain 184 bulk RNA-seq samples in total. It is known that the quantification, gene coverage, and file format of the author-provided processed gene expression matrixes varied from dataset to dataset. Therefore, to reduce laboratory bias and systematic errors, we calculated and corrected the expression values of each sample by starting from its raw sequencing data. First, the raw sequencing data were downloaded from the SRA database and transformed into FASTQ format by SRA-Toolkit (https://trace.ncbi.nlm.nih.gov/ Traces/sra/sra.cgi?view = software). Adapters and low-quality reads were automatically removed by fastp software (Chen et al., 2018) to control the quality of reads. After quality control, the clean reads were aligned to mouse genome (GRCm38) using STAR (Dobin et al., 2013) (version 2.7.3a with default parameter and GENCODE version M24 GTF gene annotation), and the gene expression quantification of each sample was performed by using the RSEM software (version 1.3.1, with default parameter) (Li and Dewey, 2011). The gene expression read counts from each sample were merged into the gene expression matrix by the Perl script modified from RSEM software.

An unexpected, prominent variation between samples from the same disease condition group but different datasets was observed (Supplementary Figure S1), derived by principal component analysis (PCA), where the samples were more likely to be clustered according to the datasets rather than to the disease conditions. Therefore, the batch effect emerged as a serious problem before any further analysis based on the gene expression matrix. To correct these batch differences, the whole gene expression matrix which contained all samples was first processed by quantile normalization, and then the normalized expression matrix was submitted to batch correction through the popular ComBat tool (Johnson et al., 2007). To minimize the batch effects and retain biological variations between samples, the batch covariate and type covariate were both considered when running the ComBat tool. Nevertheless, there were still some outlier datasets that did not cluster according to disease condition after correction. After removing these outlier samples, the corrected bulk RNA-seq expression matrix covering 110 samples from 12 datasets (Supplementary Table S1) was obtained, which included disease model samples in MI, DCM, HCM, and ARVC and their corresponding healthy samples.

### Preparation of Heart Single-Cell Expression Reference Dataset

As for the reference scRNA-seq data, we first downloaded the single-cell expression data of mouse hearts from Tabula Muris Consortium (Tabula Muris, 2018). To accurately annotate the cell types, we used the Seurat R package (v3.2.0) (Satija et al., 2015) to perform unsupervised clustering of the single-cell read count profiles. The read counts from each cell were divided by the total read count of that cell and then were multiplied by a scaling factor of 10,000 and log-transformed. To improve the clustering stability, genes detected in less than 10 cells were excluded from the downstream analysis. We performed PCA on the normalized expression matrix using highly variable genes identified by the *FindVariableGenes* function of Seurat. Following the result of the PCA, the top significant principal components were selected for clustering, with a resolution parameter equal to 0.5. The cell clusters were visualized using t-distributed stochastic neighbor embedding method, and their corresponding markers were listed using the *FindAllMarkers* function of Seurat. Finally, the cell type or subtype of each cluster was annotated based on the known cell type markers from the CellMarker database (Zhang X. et al., 2019). For healthy heart samples from Tabula Muris Consortium, six cell types were annotated by this procedure, and four of them were used to further build a signature matrix, including cardiomyocyte, endothelial cell, fibroblast, and smooth muscle cell.

To better understand the disease-related cell abundance alteration, cell-type-specific pathway alteration, and/or cell subpopulation alteration, we also introduced disease-related scRNA-seq datasets from the GEO database as the alternative scRNA-seq references. These alternative disease-related GEO references include the following:

(1) MI-related scRNA-seq dataset (GSE120064) of Ren et al.: This reference datasets covered cardiomyocytes, endothelial cells, fibroblasts, smooth muscle cells, and macrophages in the MI mouse model’s heart tissues (Ren et al., 2020).
(2) HCM-related scRNA-seq dataset (GSE129175) of Zhang et al.: This reference datasets covered cardiomyocytes, endothelial cells, fibroblasts, smooth muscle cells, and macrophages in the HCM mouse model’s heart tissues (Zhang Y. et al., 2019).
(3) MI-related, fibroblast subpopulation-annotated scRNA-seq dataset (GSE132144) of Ruiz-Villalba et al.: This reference datasets contained six major subpopulations of fibroblasts (sp1 to sp6; some minimal subpopulations were not considered) in the MI mouse model’s heart tissues. This reference was specifically used to analyze the alteration of fibroblast subpopulations in different disease conditions (Ruiz-Villalba et al., 2020).

### Estimation of Cell Type Fraction and Reconstruction of Cell-Type-Specific Gene Expression Profiles

CIBERSORTx is a new machine-learning-based method for estimating the relative abundance of cell types and cell-type-specific expression from the bulk RNA-seq data. To achieve this, the initial step is to construct a signature matrix featuring the gene expression characteristics of each cell type. We here considered two sources of cell type annotations. First, the aforementioned scRNA-seq reference data processed from Tabula Muris reference or the alternative disease-related GEO references were used for the four major cell types of the heart, which was prepared and formatted as a standard signature matrix by the Create Signature Matrix analysis module of CIBERSORTx with suggested parameters. Second, to further investigate the immune microenvironment of heart tissues of different diseased states, the built-in LM22 immune cell signature matrix was used to estimate the immune cell infiltration and expression. To observe consistency with other mouse expression matrixes, we converted the human gene symbols of LM22 into the corresponding mouse homologous gene symbols before calculation. Then, the two prepared signature matrix files along with the batch-corrected bulk RNA-seq gene expression matrix were submitted to the Impute Cell Fraction and Impute Cell Expression analysis modules of CIBERSORTx to estimate the relative abundance and expression for each of the 26 cell types in the signature matrixes, respectively. When imputing cell fraction, the S-mode was applied to remove batch effect between signature matrix and mixture samples. When imputing gene expression, high resolution mode was used to further enable the estimation of cell-type-specific gene expression profiles for each of the 110 bulk RNA-seq samples. A gene subset file which covers a list of 18,584 genes from the bulk expression file was used as input for analysis of as many genes covered by the bulk transcriptomes as possible.

### Differentially Expressed Gene Screening and Functional Enrichment Analysis

The reconstructed cell-type-specific gene expression matrixes allow the screening and analysis of genes differentially expressed for each cell type between disease (MI, DCM, HCM, and ARVC) and healthy (or control) samples. Taking the cell-type-specific gene expression matrix as the input, cell-type-specific DEGs were calculated by DESeq2 (Love et al., 2014), and those with adjusted *p*-value <0.05 were considered as the significant DEGs. The g:Profiler (Raudvere et al., 2019) is a web server for simultaneously performing multiple functional enrichment analyses with timely updated reference gene function annotations. To explore the potential biological functions of these cell-type-specific DEGs, the DEG lists were submitted to g:Profiler to investigate their associated significantly overrepresented (adjusted *p*-value <0.05) Gene Ontology functional terms and WikiPathways biological pathways. As for the disease gene enrichment analysis, the disease gene annotations were firstly retrieved from DisGeNET database (Piñero et al., 2017), and only “heart disease” and its child diseases terms (*e*.*g*., myocardial infarction) were retained. We adopted Fisher exact test to test the cell-type-specific heart disease gene associations. More specifically, genes related to each heart disease were treated as one gene set, and the enrichment of these disease genes in each cell-type-specific DEG set was compared to the background (*i*.*e*., all genes in the transcriptome) by Fisher exact test. For each set of cell-type-specific DEGs from MI, HCM, DCM, and ARVC, we separately calculated their significance and retained the disease gene terms satisfying the significance threshold of false discovery rate-adjusted *p*-value <0.05.

### Protein–Protein Interaction Network Analysis and Cell–Cell Communication Analysis

Protein–protein interaction network analysis was performed using GeneMANIA (Warde-Farley et al., 2010). The input genes contained the differentially expressed secretory protein genes in monocytes and the overrepresented pathway-related genes in fibroblasts. The prioritized interacting gene pairs related to the input gene list were used to build a regulatory subnetwork, which was further visualized by Cytoscape software (version 3.8.0). CellChat (Jin et al., 2021), an R-based computational analysis tool, was implemented for the analysis of cell–cell communication. The expression profiles of 26 cell types were used as the input of CellChat to construct the cell–cell communication network. Briefly, we first calculated the significant ligand–receptor pairs (LRs) between any two cell types. CellChat covered several scenarios of LRs, and only the typical *Secreted Signaling* LR sets in CellChatDB were considered. Afterwards, the LR count matrix between any two cells was constructed, and the healthy LR count matrix subtracted from the diseased LR count matrix was used to reflect changes in cell–cell communication between healthy and disease samples. The changing of cell–cell communication network was also visualized by Cytoscape.

### Myocardial Infarction Mouse Model

Ten-week-old C57BL6/J mice were used in our experiments. The mice were first anesthetized by isoflurane inhalation and intubated with an endotracheal cannula. As for the myocardial infarction group, intercostal thoracotomy was performed to expose the heart, followed by ligation of the left anterior descending coronary artery. As for the sham-operated mice, they underwent the same surgical incision without ligation. All mice were then assessed by ultrasound imaging to ensure the success of disease modeling. All animal experiment protocols complied with the Animal Management Rules of the Ministry of Health of the People’s Republic of China and the Guide for the Care and Use of the Laboratory Animals of Peking University and were approved by the Laboratory Animal Ethics Office of Peking University Biomedical Ethics Committee (LA2020337).

### Flow Cytometry Assay

At 1 or 4 weeks after ligation or sham surgery, hearts were harvested from the euthanized mice and perfused with 20 ml of cold phosphate-buffered saline (PBS). The hearts were then minced and digested with shaking for 30 min at 37°C in RPMI 1640 medium containing collagenase I (450 U/ml, Sigma, St. Louis, USA), collagenase XI (125 U/ml, Sigma, St. Louis, USA), DNase I (60 U/ml, Roche, Basel, Switzerland), and hyaluronidase (60 U/ml, Harveybio, Beijing, China) enzymes. The digested tissues were passed through a 40-μm filter, centrifuged at 1,600 rpm for 6 min, and resuspended in PBS containing 2% fetal bovine serum, followed with antibody staining. The antibodies used in the experiment are presented in Supplementary Table S2.

Single-cell suspensions were labeled with antibodies for 30 min at 4°C and washed in FACS buffer. Flow cytometry analysis was performed on LSRFortessa (BD Biosciences), and the results were analyzed with FlowJo software. Analysis of significant differences on cell counts in flow cytometry between healthy and MI samples was performed using Student’s *t*-test. The differences on calculative composition were analyzed using Mann–Whitney test.

## Results

### Overview of the Bulk and Reconstructed Cell-Type-Specific Expression Profiles of Mouse Hearts From Cardiac Disease Models

**FIGURE 2:**
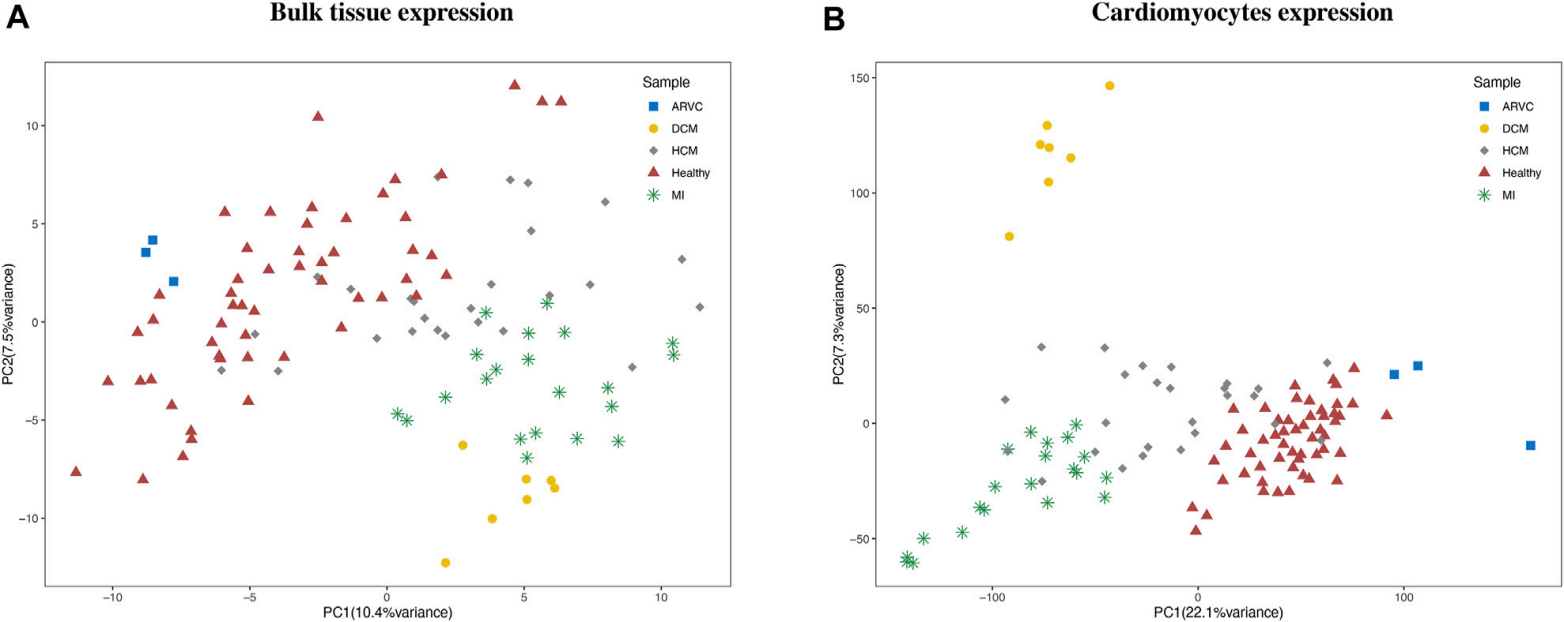
Clustering of bulk RNA-seq expression profiles and reconstructed cardiomyocyte expression profiles. The principal component analysis clustering results using **(A)** bulk expression profile and **(B)** reconstructed cardiomyocyte expression profiles are labeled according to the disease condition of each sample. ARVC, arrhythmogenic right ventricular cardiomyopathy; DCM, dilated cardiomyopathy; HCM, hypertrophic cardiomyopathy; MI, myocardial infarction.

Multiple bulk RNA-Seq data could be retrieved by searching the GEO database (Barrett et al., 2013) with cardiac disease-related keywords. However, the author-provided gene expression matrixes which are readily downloadable on GEO are not suitable for our integrative analysis purpose because of several pronounced problems, like inappropriate sample source (*e*.*g*., blood samples), discrepancy of expression quantification methods, and prominent batch effects. Therefore, we instead started from the raw sequencing data and applied an extensive and carefully standardized pipeline for data collection and processing (see “*Materials and Methods*”). As a result, we assembled a batch-corrected bulk RNA-seq expression matrix covering 110 samples from 12 independent datasets. Four types of cardiac disease samples, including MI, DCM, HCM, and ARVC, and their corresponding healthy controls were included in this matrix. We noted that a reasonable PCA clustering result could be observed based on this corrected expression matrix, where samples that belong to the same disease group were clustered together well even with the variations in source datasets and disease modeling approaches (Figure 2A). This PCA result indicates a successful reduction of technical bias and retention of biological signatures in the bulk expression matrix produced from our standardized data processing pipeline.

To reconstruct cell-type-specific gene expression profiles, the gene expression characteristics should be first extracted from a comprehensive scRNA-seq reference data. To this end, the scRNA-seq reference data of mouse heart was obtained from Tabula Muris Consortium (Tabula Muris, 2018). By cell clustering and marker gene annotation, we identified four major cell types (Supplementary Figure S2) of heart from this data, including cardiomyocyte (with Actc1 and Actn2 markers), fibroblast (Col1a2 and Col3a1markers), endothelial cell (Egfl7 and Emcn markers), and smooth muscle cell (Rgs5 and Acta2 markers). Previous studies have also confirmed these four types as the main cell types in heart tissue (Litviňuková et al., 2020; Wang et al., 2020), while the macrophages and erythrocytes were not consistently detected in other heart scRNA-seq datasets and arouse concern of blood contamination and thus were not considered in the subsequent analyses. Therefore, the scRNA-seq expression matrix for the four major cell types was used as the representative reference for further analysis. Based on this reference, the input bulk RNA-seq expression matrix can be dissected in a cell-type-specific manner *via* the recently established CIBERSORTx computational approach (Steen et al., 2020). More specifically, for each bulk RNA-seq sample, the relative fraction and the gene expression profile of each cell type was calculated by CIBERSORTx. To preliminarily assess the reliability of the reconstructed cell-type-specific expression matrix, we performed PCA based on the expression matrix of cardiomyocytes (Figure 2B). The clustering result revealed that the cardiomyocyte expression profiles were clearly clustered according to the disease condition, demonstrating the recapitulation of *bona fide* biological characteristics of cardiomyocytes in cardiac diseases by the reconstructed expression matrix.

### Fibroblast Fraction Was Significantly Increased in Mouse Heart in Myocardial Infarction and Dilated Cardiomyopathy

**FIGURE 3:**
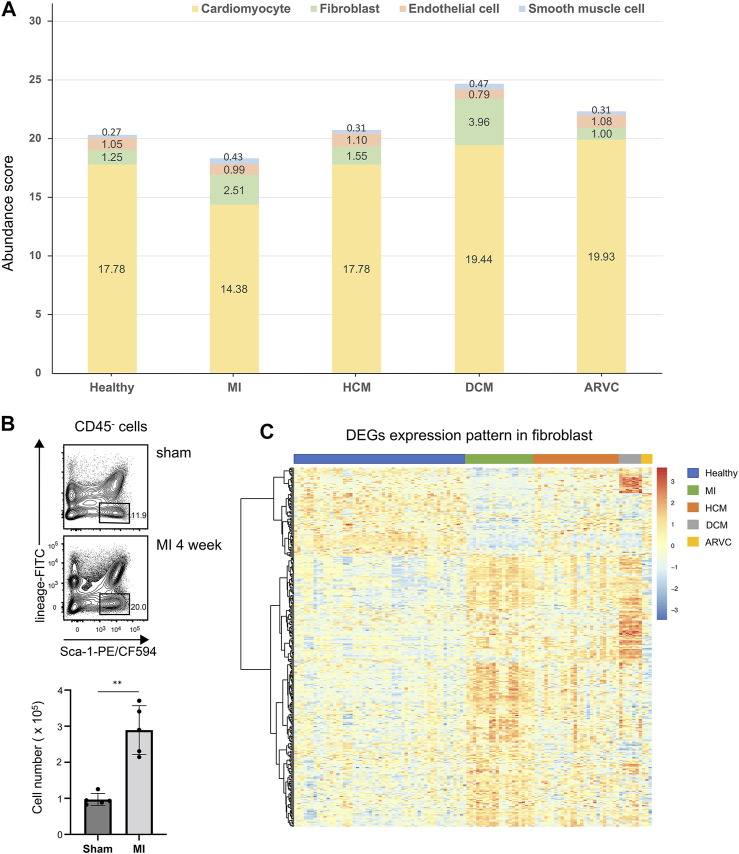
Cell abundance analysis of four major cell types of reconstructed heart transcriptomes across various cardiac disease conditions. **(A)** Bar plots showing the variation in the proportions of the four major cell types across different cardiac diseases. Each color represents a specific cell type. **(B)** Flow cytometry assay verifying that fibroblasts are increased in mouse heart tissues of myocardial infarction at the f4th week after model preparation. The top panel shows a representative sample of the flow cytometry, and the lower panel is the statistical chart (*n* = 5, *p* = 1.185 × 10^–3^, Student’s *t*-test). Gating strategy: CD45.2- lineage (TER119 + CD31)- Sca-1+. **(C)** Heat map showing the overall differentially expressed gene expression pattern of fibroblasts across different disease conditions.

We first investigate the alteration of cell composition in cardiac disease condition based on the estimated cell abundance of CIBERSORTx. The average abundances of the four major cell types in different sample groups are shown in Figure 3A. According to the results, cardiomyocytes which constitute over 80% of these four main cell types exhibit the highest abundance in all sample groups. Following cardiomyocytes, the next abundant cell types are endothelial cells and fibroblasts in healthy samples. Interestingly, a recent study (Pinto et al., 2016) based on new genetic tracers and enhanced flow cytometry techniques has confirmed endothelial cells as the most abundant cell population other than myocytes, while other studies considered that fibroblasts are more abundant than endothelial cells (Banerjee et al., 2007; Bergmann et al., 2015). On the one hand, our results also support the considerable proportions of endothelial cells and fibroblasts in heart tissues, where their abundances are largely comparable in healthy heart tissues. On the other hand and more importantly, by comparing the estimated abundances between the disease and healthy sample groups, a substantial alteration of the relative abundances of these two cell types can be observed.

As noted above, the comparison also indicates alteration of cell composition in pathological conditions. In MI samples, the abundances of cardiomyocytes are significantly decreased compared to the healthy samples (*p* < 0.01, Mann–Whitney test). One straightforward explanation of this observation would be the hypoxia-induced cell death of a vast number of cardiomyocytes in proximity to the damaged area in MI (Frangogiannis, 2015; Martín-Fernández and Gredilla, 2016). On the contrary, a significant elevation of fibroblast abundance can be observed not only in MI samples (*p* < 0.01, Mann–Whitney test) but also in DCM samples (*p* < 0.01, Mann–Whitney test), indicating the cardiac fibrosis progression in these disease conditions. To verify these results, we adopted two other scRNA-seq-based transcriptome reconstruction methods, namely, MuSiC (Wang et al., 2019) and Bisque (Jew et al., 2020), to estimate the cell type abundance using the same single-cell reference. It is noteworthy that, unlike CIBERSORTx, MuSiC and Bisque can only calculate the cell type relative abundance (*i*.*e*., cell proportions that sum up to 100%). The estimated cell type abundance scores from MuSiC and Bisque are summarized in Supplementary Figure S3. Despite the distinguishing cellular abundance scores between the different methods, the variations of cell type abundance scores across different disease conditions are highly similar to those from CIBERSORTx—for example, the relative score of cardiomyocytes is decreasing in MI and of fibroblast is increasing in MI and DCM compared with the healthy samples (*p* < 0.01, Mann–Whitney test). Such trend is the same as that observed in the CIBERSORTx results.

To further verify this observation, we applied flow cytometric assay to compare the fibroblast abundances in healthy and MI mouse hearts. Indeed a significantly increased fibroblast fraction is observed in MI condition in comparison with the sham condition, which also confirmed our result (Figure 3B). Cardiac fibrosis is a prevalent pathophysiological process in many myocardial diseases and considered to be the end state of heart injury along with increasing fibroblasts (Kong et al., 2014). Our analysis implies that cell proliferation may not be the sole source of the increased fibroblasts—for example, in DCM samples, the average abundance of endothelial cells is decreased (*p* < 0.01, Mann–Whitney test) along with the increased fibroblasts, indicating that endothelial-to-mesenchymal transition (EndMT) would be a common event during heart fibrosis in DCM. EndMT is an intricate cellular differentiation process in which endothelial cells lose their properties and acquire mesenchymal features, and this process would give rise to the transition from endothelial cells to fibroblasts (Cheng et al., 2021). Indeed Xie *et al*. have demonstrated that EndMT might contribute to myofibroblast recruitment in human DCM, characterized by decreased endothelial markers and increased mesenchymal markers in the immunofluorescence co-localization analysis (Xie et al., 2018). They also found that several cells expressed both mesenchymal and endothelial markers, which occurred exclusively in the cardiac sections of DCM patients but not in normal cardiac samples. Moreover, Zhang *et al*. has observed a significantly elevated EndMT in DCM rats, and this process can be suppressed through inhibition of TGF-β/ERK signaling (Zhang C. et al., 2019). In a mouse model of cardiac fibrosis, transforming growth factor-beta 1 (TGF-beta1) can induce the endothelial cells to undergo EndMT, whereas bone morphogenetic protein 7 can preserve the endothelial phenotype (Hong et al., 2018; Piera-Velazquez and Jimenez, 2019).

### Fibroblast-Related Signaling Pathways Are Activated Under Various Cardiac Diseases

**FIGURE 4:**
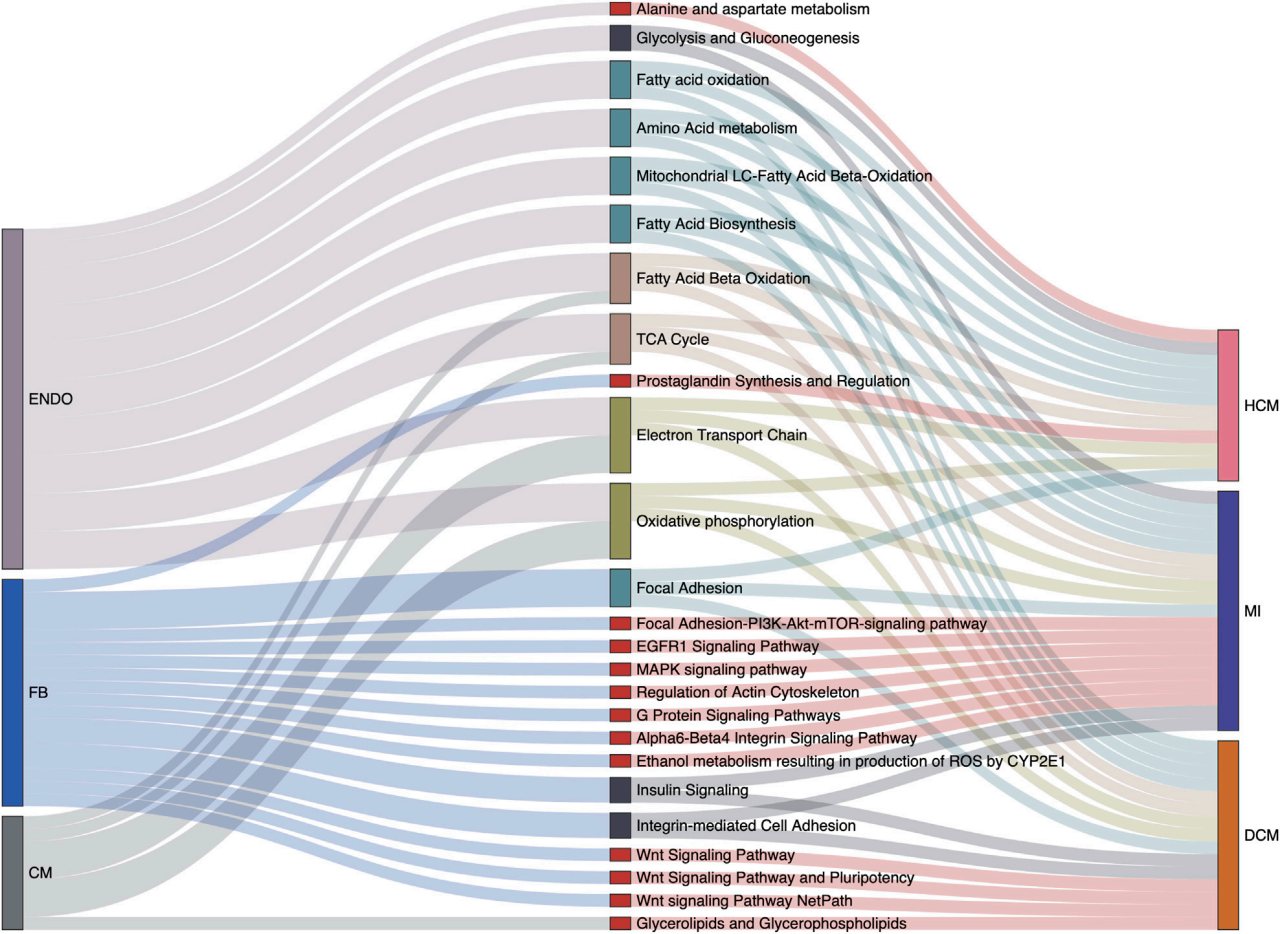
Cell type-specific overrepresented pathways associated with various cardiac disease conditions. Relations among cell types, signaling pathways from the WikiPathways database, and disease conditions are summarized in the Sankey plot. Only the pathways significantly enriched at least among one of the cell-specific differentially expressed gene (DEG) sets in one disease condition are considered. Besides this, pathways actually describing disease gene sets are excluded to avoid confusion. The width of a link correlates with the strength of the relation (*i*.*e*., the relative counts of pathway genes in the DEGs of a specific cell type or a specific disease condition).

Varied gene expression patterns of fibroblasts can be observed between different disease conditions (Figure 3C), suggesting the plausible functional heterogeneity of fibroblasts in different cardiac diseases. To further explore the related biological functions in each individual cell type, the cell-type-specific DEGs were determined following the method described above (“Materials and Methods”). Among the four major cell types, fibroblasts indeed show the largest amount of DEGs, followed by cardiomyocytes and endothelial cells, while DEGs can hardly be detected in smooth muscle cells (Supplementary Figure S4 and Supplementary Table S3). Based on these identified DEGs, WikiPathways and Gene Ontology (GO) enrichment analyses were performed for each cell type in each specific disease condition. The results of the WikiPathways enrichment analysis are summarized in Figure 4 and Supplementary Table S4. Few significant pathways are obtained from ARVC DEGs for any cell types and smooth muscle cell DEGs for any disease conditions (which is also resulting from the limited DEGs identified in these cases, as shown in Supplementary Table S3), so the functional enrichment analysis results for these DEGs are not presented. In summary, the pathways enriched in cardiomyocytes are largely shared across disease conditions (Figure 4), where the pathways related to mitochondrial energy metabolism are most prominent, such as electron transport chain, oxidative phosphorylation, fatty acid beta oxidation, and tricarboxylic acid cycle. Moreover, the GO enrichment term analysis also provides signatures associated with mitochondrial dysfunction, like mitochondrion organization and electron transfer activity (Supplementary Figure S5). The mammalian heart must contract incessantly to sustain life, which is heavily dependent on adenosine triphosphate (ATP) production and consumption (Doenst et al., 2013). The cardiomyocytes had consumed more than 90% ATP generated by the mitochondria, and thus the cardiomyocyte function requires proper mitochondrial energy metabolism. However, our analysis suggests mitochondrial dysfunction in cardiomyocytes accompanied with the development of cardiac diseases like MI, HCM, and DCM, which could be also confirmed by previous experimental researches (Peoples et al., 2019). In addition to cardiomyocytes, endothelial cells also have many enriched pathways related to energy production, suggesting its similarity with cardiomyocytes in cellular response to disease state.

Unlike cardiomyocytes and endothelial cells, the associations with energy metabolism are much less prevalent for fibroblasts. Instead we find that Wnt signaling pathway is specifically enriched in fibroblasts under DCM condition. Wnt signaling is a complex collection of signal transduction pathways, and the aberrant regulation of the Wnt signaling pathway is associated with many diseases, such as cancer, degenerative diseases, and also cardiovascular diseases (Nusse and Clevers, 2017; Foulquier et al., 2018; Lietman et al., 2018). The significant enrichment of the Wnt pathway in fibroblasts suggests its important role in the occurrence and development of DCM, and indeed one recent study has described that canonical WNT/β-catenin signaling activity is impaired in DCM, and reactivation of its activity can improve cardiac contractility and ameliorate intraventricular conduction defects (Lu et al., 2016). The MAPK signaling pathway is another specifically enriched pathway in fibroblasts in MI, which is also recently proven to modulate the apoptosis of myocardial cells in acute MI heart (Zhang et al., 2020). The GO term enrichment analysis in fibroblasts is also related with MAPK cascade, actin cytoskeleton organization, and cell adhesion molecular binding (Supplementary Figure S5), which also supports the pathway enrichment result. Notably, several pathways mapped to fibroblasts have not been reported, including the EGFR1 signaling pathway, the insulin signaling pathway, and the alpha6-beta4 integrin signaling pathway. The involvement and mechanisms of these pathways in cardiac fibrosis and cardiac pathogenic process would be an interesting topic for further investigation according to our cell-type-specific pathway analysis.

Furthermore, to establish a relationship between cell types and the known disease genes of cardiac diseases, we performed a disease gene enrichment analysis for each cell-type-specific DEG set (see “Materials and Methods”). The results (Supplementary Figure S6) demonstrate the diversity of cell-type-specific associations with disease terms between different disease conditions—for example, in MI, the fibroblasts and endothelial cells are associated with the *myocardial infarction* term. As for HCM and DCM, the HCM-associated cell types include cardiomyocyte and endothelial cells, while the DCM-associated cell types include cardiomyocytes, fibroblasts, and endothelial cells. This observation indicates their essential role in the occurrence of corresponding diseases and would be helpful for further cell-type-specific disease mechanism investigations.

### Transcriptome Reconstruction Using Alternative Disease-Related scRNA-Seq References Reveals the Upregulation of a Specific Fibroblast Subpopulation in Myocardial Infarction and Dilated Cardiomyopathy

**FIGURE 5:**
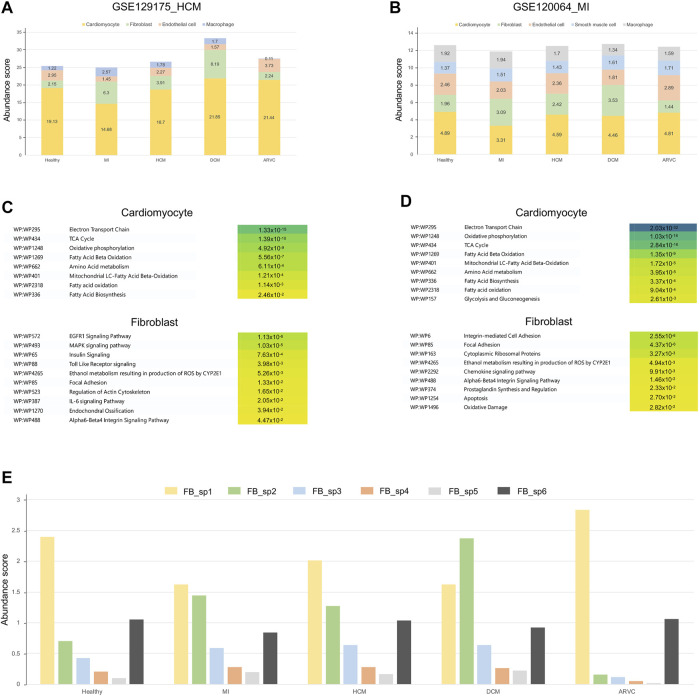
Overview of transcriptome reconstruction results using disease-related scRNA-seq datasets from myocardial infarction (MI) and hypertrophic cardiomyopathy (HCM) heart samples as the alternative references. **(A)** Bar plot showing the cell type compositions estimated from the HCM scRNA-seq references (GSE129175). **(B)** Bar plot showing the cell type compositions estimated from the MI scRNA-seq references (GSE120064). **(C)** The top enriched pathways of differentially expressed genes (DEGs) in cardiomyocytes and fibroblasts using the HCM scRNA-seq references. The corrected *p*-values are shown on the colored boxes. Pathways actually describing disease gene sets are excluded to avoid confusion. **(D)** The top enriched pathways of DEGs in cardiomyocytes and fibroblasts using the MI scRNA-seq references. The corrected *p*-values are shown on the colored boxes. Pathways actually describing disease gene sets are excluded to avoid confusion. **(E)** The abundance of fibroblast subpopulations (FB_sp1 to FB_sp6) estimated from the fibroblast subgroup-annotated MI-related scRNA-seq reference (GSE132144) across different cardiac disease conditions.

The above-mentioned analyses were based on the single-cell references derived from healthy samples. However, scRNA-seq data derived from the corresponding heart diseases could provide additional valuable information. To this end, we introduced two other public scRNA-seq datasets derived from MI (Ren et al., 2020) and HCM (Zhang Y. et al., 2019) samples as the alternative, disease-related scRNA-seq references. By reconstruction of bulk transcriptome profiles using these two reference datasets, we have re-estimated the cell type’s abundances and cell-type-specific pathways across different disease conditions (Figures 5A,B). Generally, the overall cell abundances and the overrepresented pathways in DEGs are similar between the results based on healthy single-cell reference and those based on the alternative disease-related single-cell references—for example, the abundance of cardiomyocytes decreases in MI and that of fibroblasts increases in MI and DCM along with a decrease of endothelial cells (*p* < 0.01, Mann–Whitney test). Furthermore, the disease-reference-based results from a cell-type-specific pathway analysis also recapitulate energy metabolism-related pathways of cardiomyocytes and several overrepresented pathways (EGFR1 signaling pathway, MAPK signaling pathway, focal adhesion, alpha6-beta4 integrin signaling pathway, and regulation of actin cytoskeleton) in fibroblasts in MI (Figures 5C,D). On the other hand, we also found several pathways which are not represented in our previous healthy reference-based analysis, like IL-6 signaling pathway and oxidative damage in fibroblasts. It is well accepted that IL-6 is mainly secreted by fibroblasts in the heart and can promote CF proliferation by promoting EndMT in ECs (Zhang et al., 2021). This observation indicates that combining those different datasets is helpful for a more comprehensive knowledge associated with cardiac diseases.

As shown above (Figure 3A and Figures 4A,B), fibroblasts were suggested to be an important participant of the transcriptome alterations of heart tissue in multiple disease conditions like MI and DCM. It is interesting to investigate if particular subpopulation(s) of fibroblasts contribute more to such alterations. To further identify which subpopulation of fibroblasts is activated in the disease conditions, we further introduced a fibroblast subpopulation-annotated scRNA-seq data (Ruiz-Villalba et al., 2020) as the alternative reference. Six major fibroblast subpopulations (*i*.*e*., FB_sp1 to FB_sp6) were annotated in this MI-related, fibroblast subpopulation-annotated scRNA-seq reference. As shown in Figure 5E, FB_sp2 that is featured in the high expression of Cthrc1 is observed to be increased in MI, DCM, and HCM (*p* < 0.01, Mann–Whitney test). Remarkably, FB_sp2 has been experimentally verified to be increased in the infarcted myocardium (Ruiz-Villalba et al., 2020). FB_sp3 (Efhd1 and Cd248 highly expressed) is another fibroblast subpopulation showing an elevated abundance in MI, DCM, and HCM (*p* < 0.01, Mann–Whitney test). Other subpopulations, like FB_sp1 (Hsd11b1 highly expressed), is the main subtype in ARVC where the amount of FB_sp2 is very few. Taken together, our results suggest that FB_sp2 may serve a crucial role in other cardiac diseases besides MI, and other subpopulations like FB_sp1 and FB_sp3 may also be associated with some specific cardiac diseases.

### Analysis of the Cardiac Immune Microenvironment Suggests the Involvement of Monocytes in the Development of Myocardial Infarction

**FIGURE 6:**
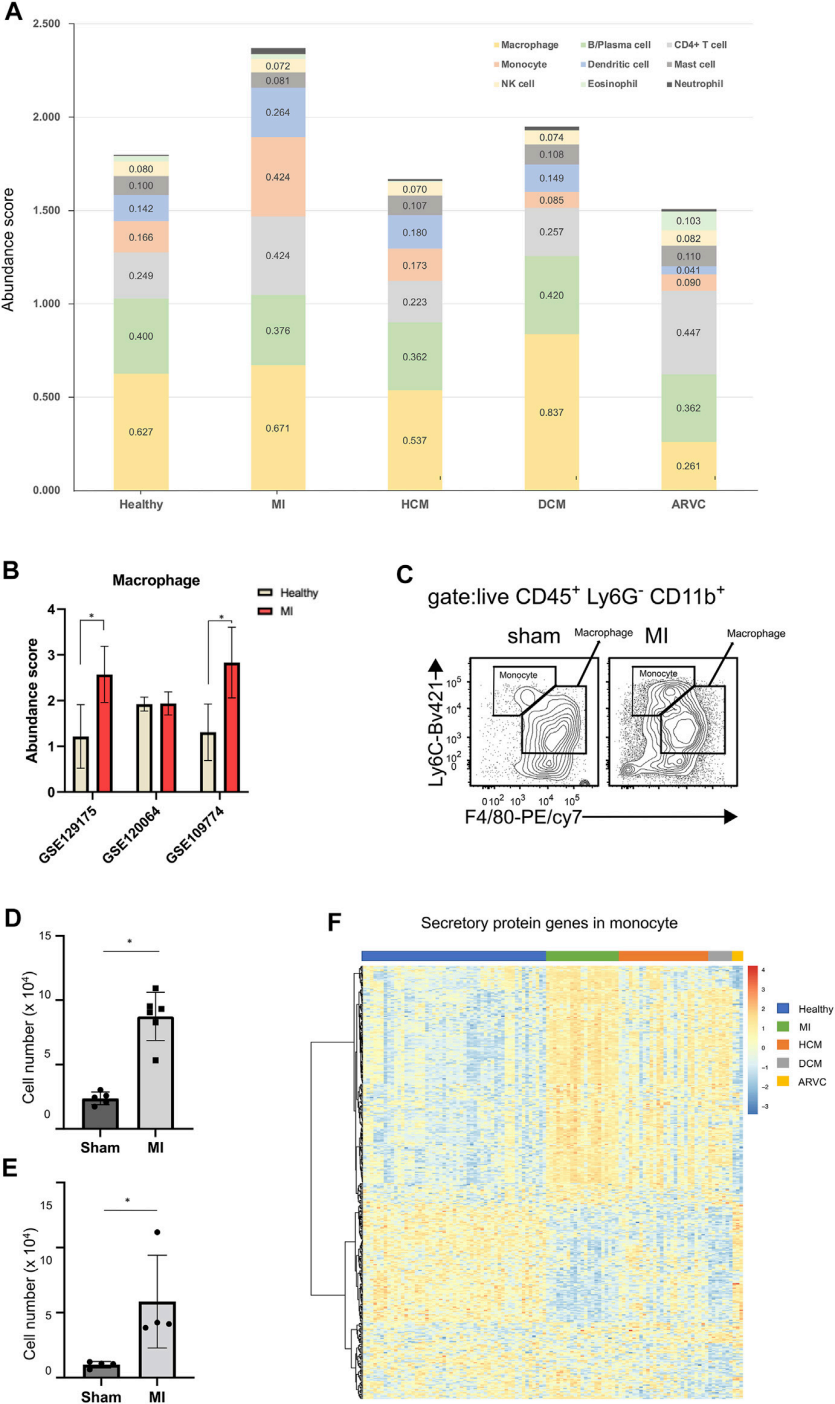
Immune cell infiltration analysis of reconstructed heart transcriptomes across various cardiac disease conditions. **(A)** Bar plots showing the variation in the proportions of the 22 immune cell subtypes (LM22 reference) across different cardiac diseases. Each color represents a specific immune cell subtype. **(B)** Comparison of macrophage abundances estimated by using the alternative scRNA-seq references. **(C)** Flow cytometry assay verifying that macrophages and monocytes are increased in mouse heart tissues of myocardial infarction (MI) at the 1st week after model preparation. The result shows a representative sample of flow cytometry. Gating strategy for macrophage: CD45.2^+^ Ly6G^−^ CD11b^+^ F4/80^+^; gating strategy for monocyte: CD45.2^+^ Ly6G^−^ CD11b^+^ F4/80^−^. **(D)** Statistical chart (*n* = 5, *p* = 2.368 × 10^–3^, Student’s *t*-test) showing the macrophage increasing in MI. **(E)** Statistical chart (*n* = 4, *p* = 3.598 × 10^–2^, Student’s *t*-test) showing the monocyte increasing in MI. **(F)** Heat map showing the expression pattern of secretory protein genes in monocytes across different disease conditions.

Immune cells play crucial roles in the occurrence and development of cardiovascular diseases, and many studies have demonstrated that inflammatory response is prevalent under cardiac pathophysiological conditions (Bartekova et al., 2018). Though some inflammation processes are required for tissue repair during injury and are therefore protective (Liao et al., 2018), prolonged inflammation leads to myocardial remodeling and apoptosis of cardiomyocytes (Frangogiannis, 2014). To further compare the differences in immune cell infiltration among different disease types, we used the built-in LM22 immune cell reference of CIBERSORTx to calculate the abundance and cell-type-specific expression profile of various immune cell subtypes in mouse hearts. We find that alteration of immune cell subtype abundance is a common phenomenon across different cardiac diseases (Figure 6A)—for example, the number of macrophages increases in DCM along with reduction of monocytes (*p* < 0.01, Mann–Whitney test). It is widely accepted that monocytes are the precursor cells of macrophages and are recruited to the inflammatory site by inflammatory factors (Peet et al., 2020), and such increased number of macrophages and decreased number of monocytes would suggest that the monocytes are more likely to differentiate into macrophages in DCM.

When using the LM22 built-in immune cell reference of CIBERSORTx, the result suggests that the abundance of monocytes increases, but the abundance of macrophages remains almost unchanged. However, several previous studies have reported upregulated macrophages in MI (King et al., 2017; Farbehi et al., 2019). In order to explore the reason of this discrepancy, we used three other scRNA-seq data (GSE129175 from HCM, GSE120064 from MI, and GSE109744 from healthy samples) as the alternative references to estimate macrophage abundance in MI (Figure 6B). We found that two out of the three results indicate an elevated macrophage abundance in MI. Furthermore, our result of the flow cytometry assay confirms the significantly elevated macrophage abundance in mouse hearts in the MI group compared with the sham group (Figures 6C,D). This result indicates that the external scRNA-seq data is a better choice for analyzing macrophage transcriptomes than the built-in LM22 references. However, most other immune cell (sub) types are not covered by external scRNA-seq data, and an analysis based on the LM22 reference is able to provide useful information, as exemplified below. A previous study has speculated that monocytes can serve as critical mediators of the inflammatory response and also mediate cardiac repair in the development of MI (Heidt et al., 2014). Therefore, we assume that not just differentiated macrophages but also monocytes can serve as primary myeloid cells that contribute to MI. Although the GEO scRNA-seq datasets did not cover reference single-cell expression profiles for monocytes, the LM22-based analysis indicates the increased monocyte fraction in MI (Figure 6A). A further flow cytometry assay also confirmed the significantly elevated monocyte abundance in mouse hearts in the MI group compared with the sham group using the gating strategy that could distinguish monocytes from macrophages (Figures 6C,E). Since monocytes usually play a biological role through secretory proteins (Liu et al., 2018; McKiernan et al., 2020), we further explored the expression pattern of secretory proteins in monocytes (Figure 6F). Indeed the heat map shows that the expression characteristics of the secreted proteins in MI are different from those of the healthy and other diseased states.

Certainly the above-mentioned analysis is not merely limited to monocytes but covers 22 representative immune cell subtypes. Results about other immune cell subtypes would also provide helpful clues to investigate the immune involvement in cardiac diseases. An example is ARVC, where the abundance of T cells and eosinophils increases while the abundance of macrophages decreases (*p* < 0.01, Mann–Whitney test). A clinical investigation has shown that inflammatory infiltration in the ventricular myocardium of ARVC samples is associated with severe structural heart changes, and T-lymphocytes are the main infiltrated immune cell type (Campuzano et al., 2012), indicating that T cells may exacerbate the progress of ARVC.

### Cell–Cell Communication Analysis Identified the Plausible Regulatory Network Between Monocytes and Fibroblasts

As monocytes are elevated in myocardial infarction along with proliferation of fibroblasts, we speculate that monocytes may regulate cardiac fibrosis through secretory-protein-mediated cell–cell communication. To explore this possibility, we first analyzed the possible protein–protein interaction (PPI) between the differentially expressed secreted proteins from monocytes and the overrepresented pathway-related proteins. The GeneMANIA PPI network analysis confirms the wide interactions between these two groups of proteins (Supplementary Figure S7), supporting the hypothesis that several proteins can be secreted by monocytes to regulate the function pathways in fibroblasts during the pathological cardiac fibrosis process.

**FIGURE 7:**
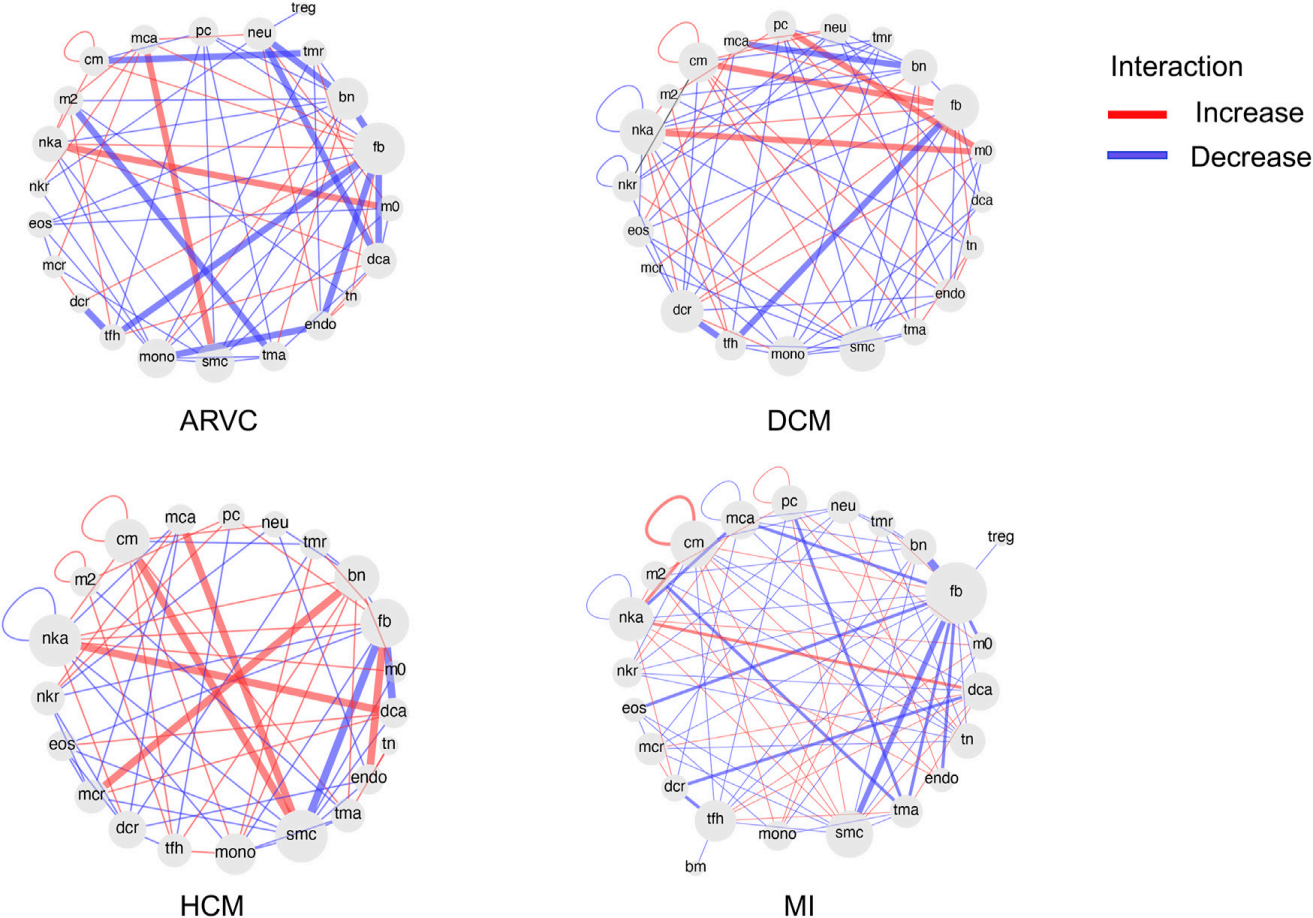
Differential cell–cell communication network based on ligand receptor analysis. For each disease condition, the differential cell–cell communication network in comparison with the healthy controls is shown. Each cell type is shown as a node in the network. The red links between nodes represent enhanced interactions between two cell types in the disease condition, while the blue links represent reduced interactions. The abbreviations of cell types are denoted as follows: cm, cardiomyocyte; mca, mast cell activated; pc, plasma cell; neu, neutrophil; treg, T cell regulatory; tmr, T cell memory resting; bn, B cell naïve; fb, fibroblast; m0, macrophage M0; dca, dendritic cell activated; tn, T cell naïve; endo, endothelial cell; tma, T cell activated; smc, smooth muscle cell; mono, monocyte; tfh, T cell follicular helper; dcr, dendritic cell resting; mcr, mast cell resting; eos, eosinophil; nkr, NK cell resting; nka, NK cell activated; nkr, NK cell resting; m2, macrophage M2; bm, B cell memory.

We further extended the cell-to-cell communication analysis to all of the 26 cell types (4 major cell types + 22 immune cell subtypes) covered in this study. We used CellChat to calculate changes in cell-to-cell communication patterns for each disease type (Figure 7). In line with the cell abundance and functional pathway analyses, for most cases, fibroblasts are at the center of the cell communication network, especially in MI and ARVC, suggesting its central role in the development of cardiac diseases. In addition, immune cells, such as activated NK cells, show strong connections with many other cell types in HCM, DCM, and MI. A recent study found that NK cells play a significant role in repairing injured tissue and maintaining tissue homeostasis (Ong et al., 2017). NK cells may function in a cardiac immune environment directly through receptor–ligand interactions or indirectly through cytokine secretion. One recent study has demonstrated that NK cells can not only protect against the development of cardiac fibrosis by limiting collagen formation in cardiac fibroblasts and by preventing the accumulation of specific inflammatory populations in the heart but also prevent monocrotaline-induced endothelial damage (Notas et al., 2009). In our result, activated NK cells enhance the interaction with macrophage in DCM and ARVC and show strong interactions with dendritic cells in MI and HCM. Indeed NK cells have been reported to accelerate the maturation of macrophages and dendritic cells, indicating its role in altering the cardiac immune microenvironment, which again suggests that the cell–cell communication analysis would be a reasonable proxy for future research of cardiac disease-associated immune microenvironment shifts.

## Discussion

In order to comprehensively analyze and compare the differences of cell-type-specific expression profiles in different kinds of cardiac diseases, we used the CIBERSORTx algorithm to impute cell type abundance and reconstruct the cell-type-specific expression profile from the integrative set of bulk tissue transcriptome data of the mouse models of four common cardiac diseases. Knowledge of the cellular composition of the heart is one of the primary information needed for researchers to understand the pathogenesis of a disease. Although researchers have visited the cardiac cellular composition in physiological conditions and described a comprehensive atlas of cellular composition (Pinto et al., 2016), the accurate knowledge of cellular composition under various cardiac disease conditions is still lacking. Based on our computational results, the composition changes in major component cell types of heart tissue are first analyzed by different methods or single-cell data references. Through comparison with literature, we note that our estimation of cell abundance alteration is largely reasonable, especially for cardiomyocytes. This result has also highlighted a significantly elevated fraction of fibroblasts, macrophages, and monocytes as a cell-type-specific signature of MI. To validate this observation, flow cytometry experiment was used to detect the abundance of monocytes, macrophages, and fibroblasts in mouse heart undergoing MI surgery.

Reconstruction of bulk transcriptomes also enables the investigations of cell-type-specific differential gene expression. Functional enrichment analysis on cell-type-specific DEGs has revealed not only shared and distinct pathways among different disease states for each cell type. Based on our results, the pathways enriched in endothelial cells and cardiomyocytes are similar across various disease conditions, which are often related to mitochondrial energy metabolism. On the contrary, fibroblasts show a distinct pathway association between different disease conditions—for example, Wnt signaling pathways are enriched in DCM samples. Previous studies have demonstrated that the protein levels of Wnt signaling were significantly increased in DCM samples, which can promote EndMT during the development of DCM (Xie et al., 2018). Focal adhesion is a type of adhesive contact between the cell and the extracellular matrix (Paluch et al., 2016), and alteration of this biological function in fibroblasts may participate in the occurrence and development of HCM. Carolina *et al*. have demonstrated that focal adhesion kinase can serve as a mediator of hypertrophy induced by an increased load (Clemente et al., 2007), while in MI, there are more signaling pathways activated in fibroblasts, such as MAPK signaling pathway, EGFR1 signaling pathway, and Alpha6-Beta4 integrin signaling pathway (Nikolopoulos et al., 2004). Some previous studies have reported that the MAPK signaling pathway was involved with the apoptosis of cardiomyocytes, and a specific inhibitor of the MAPK signaling pathway can relieve cardiomyocyte apoptosis (Zhang et al., 2017; Sun et al., 2021). Furthermore, a cell–cell communication network analysis has also highlighted fibroblasts as the dynamic communication hub for the cellular crosstalk in cardiac diseases. Therefore, a more detailed investigation on fibroblast-specific DEGs can be helpful to understand the distinctions in the molecular mechanisms of various cardiac diseases.

To explore the potential mechanism of monocyte–fibroblast communication in MI, we analyzed the PPI network between the monocyte-specific and fibroblast-specific DEGs in MI. Several secretory protein genes, like Cfh, Qsox1, Olfml3, Mmp2, Itgb1, Ccdc80, Prss23, and Plod3 are up in monocytes in the reconstructed transcriptomes. Those proteins can directly or indirectly interact with overrepresented pathway-related genes in fibroblasts, like Ywhap, Prkcd, Col4a2, and Cbl, which provides the direction for further research on the role of intercellular communication in cardiac diseases. However, it is noteworthy that these interactions mainly derived from high-throughput *in vitro* screening, and their *in vivo* communication in a diseased heart still needs to be verified.

On the other hand, the current analysis also has several limitations. First, due to the high heterogeneity of human heart transcriptomes among different datasets, we failed to correct the batch effects and therefore did not consider the human samples in this study. Moreover, improvement of the sensitivity of single-cell gene detection and completion of single cell research on different disease types are necessary to accurately elucidate the pathogenesis of cardiac diseases (Ziegenhain et al., 2017). As the case of macrophages and monocytes shown above, the LM22 built-in reference covered more cell types, and the results based on this reference correctly predicted the abundance alteration of monocytes in MI. However, the prediction result about the abundance alteration of macrophages was not correct. By contrast, mouse *in vivo* scRNA-seq datasets were more likely to correctly predict the abundance alteration of macrophages, but such datasets did not cover other immune cell types. Therefore, comparison and integration of results using different scRNA-seq references should be helpful to obtain more reasonable and accurate results. Finally, the molecular signaling pathways mentioned in the study and their relationship with cardiac diseases still need to be experimentally elucidated and verified in the future.

## Data Availability

The original contributions presented in the study are included in the article/Supplementary Material, further inquiries can be directed to the corresponding authors. The third-party bulk transcriptome datasets analyzed are available in the Gene Expression Omnibus (GEO) database (https://www.ncbi.nlm.nih.gov/geo/) with the accessions of: GSE138008, GSE138201, GSE69201, GSE75213, GSE96561, GSE101977, GSE110209, GSE112055, GSE121546, GSE129134, GSE101301, and GSE106201. The third-party scRNA-seq datasets are available in GEO database with accessions of GSE109774, GSE129175, GSE120064, and GSE132144.
