# Supplementary material for: Deciphering Cell-Type-Specific Gene Expression Signatures of Cardiac Diseases Through Reconstruction of Bulk Transcriptomes
Source: Front Cell Dev Biol. 2022 Feb 18;10:792774. doi: 10.3389/fcell.2022.792774 (PMC8894713; doi:10.3389/fcell.2022.792774)
Supplement: Supplementary file 5 [file Image1.PDF]

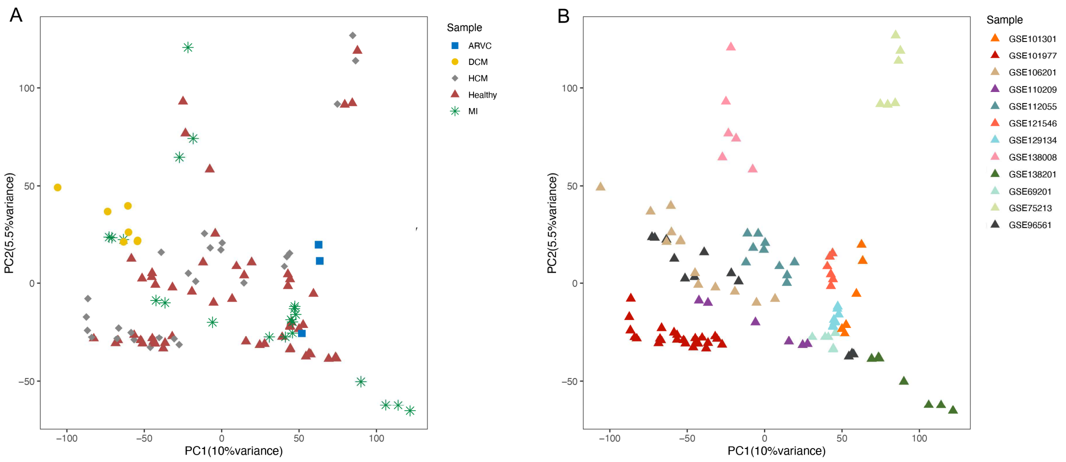

Supplementary Figure S1. Clustering analysis showing that batch effect is prominent across different datasets before correction. The sample points are colored according to (A) disease condition and (B) GEO dataset, respectively. ARVC: arrhythmogenic right ventricular cardiomyopathy; DCM: dilated cardiomyopathy; HCM: hypertrophic cardiomyopathy; MI: myocardial infarction.

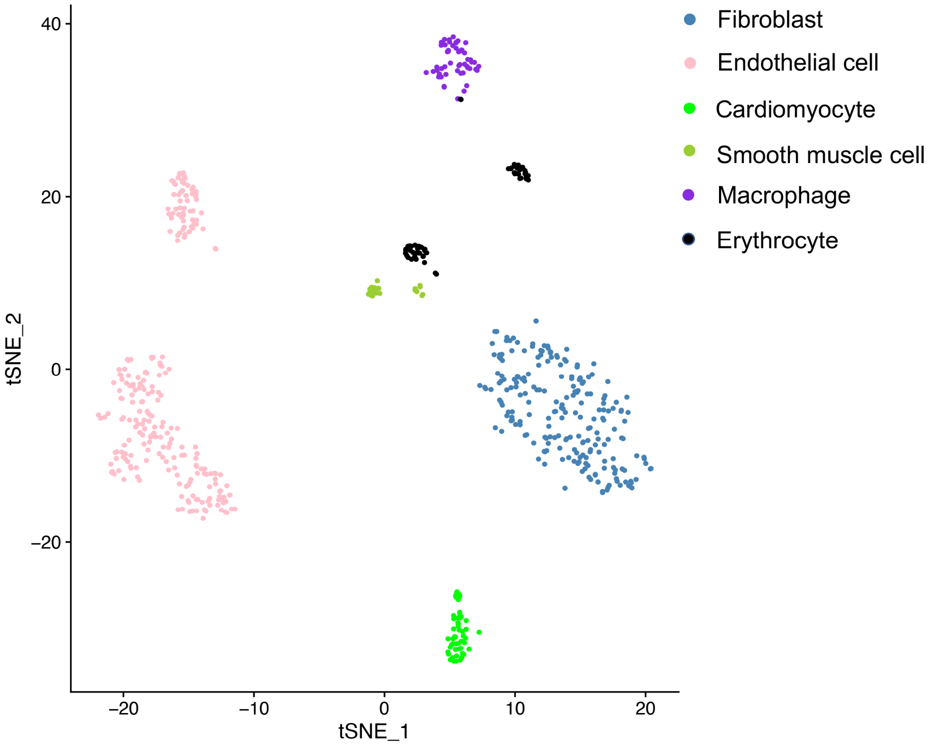

Supplementary Figure S2. tSNE visualization of scRNA-seq expression profiles covering six cell types in heart tissue from Tabula Muris Consortium.

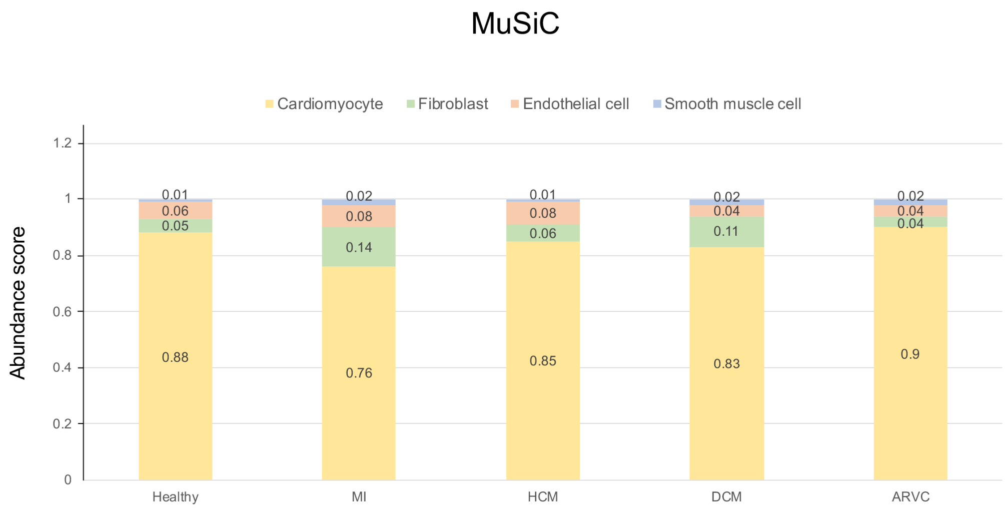

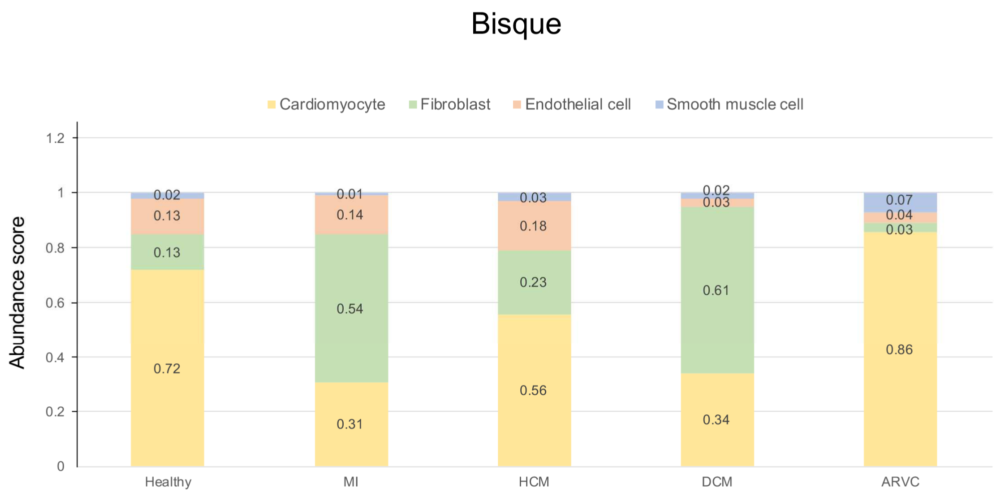

Supplementary Figure S3. Comparison of cell type proportions estimated from MuSiC and Bisque packages.

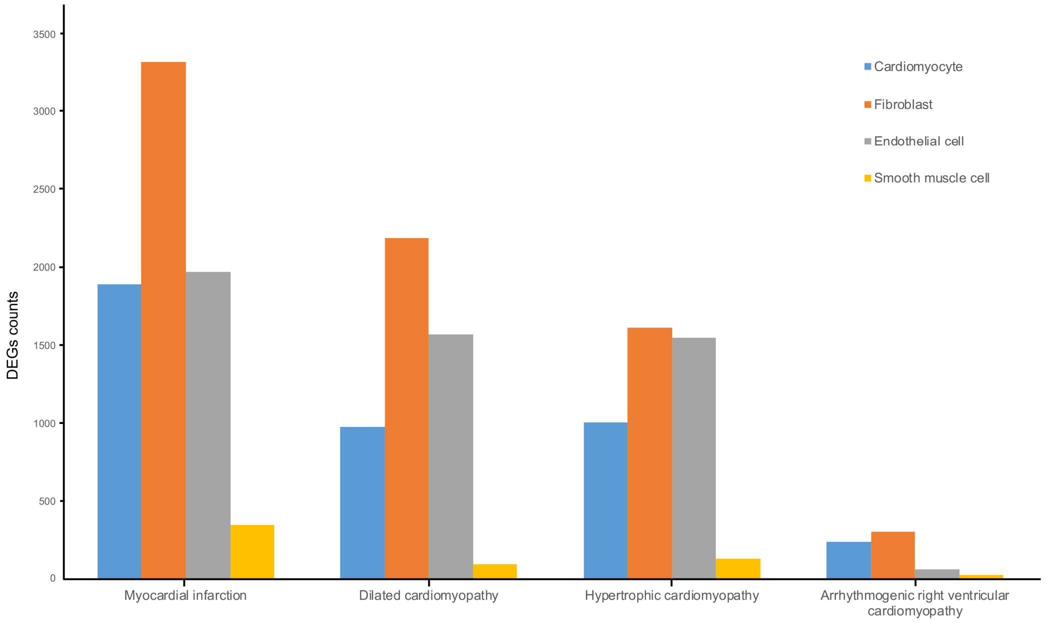

Supplementary Figure S4. Comparison of differentially expressed gene (DEG) numbers across different cardiac diseases and cell types.

	Source	Term ID	Term name	P_{adj}
Cardiomyocyte	GO:BP	GO:0007005	mitochondrion organization	$5.72x10^{-19}$
	GO:BP	GO:0006119	oxidative phosphorylation	$9.11x10^{-16}$
	GO:BP	GO:0006091	generation of precursor metabolites and energy	$8.51x10^{-15}$
	GO:BP	GO:0032981	mitochondrial respiratory chain complex I assembly	$8.82x10^{-13}$
	GO:BP	GO:0046034	ATP metabolic process	$2.58x10^{-11}$
	GO:BP	GO:0015980	energy derivation by oxidation of organic compounds	$3.94x10^{-12}$
	GO:BP	GO:0022900	electron transport chain	$1.44x10^{-8}$
	GO:MF	GO:0009055	electron transfer activity	$9.55x10^{-6}$
	GO:MF	GO:0140098	catalytic activity, acting on RNA	$2.60x10^{-5}$
	GO:MF	GO:0003955	NAD(P)H dehydrogenase (quinone) activity	$8.27x10^{-4}$
	GO:MF	GO:0016655	oxidoreductase activity, acting on NAD(P)H, quinone or similar compound as acceptor	$3.33x10^{-3}$
	GO:MF	GO:0008137	NADH dehydrogenase (ubiquinone) activity	$1.41x10^{-2}$
	GO:MF	GO:0046933	proton-transporting ATP synthase activity, rotational mechanism	$3.64x10^{-3}$
Fibroblast	GO:BP	GO:0035239	tube morphogenesis	$2.28x10^{-26}$
	GO:BP	GO:0030334	regulation of cell migration	$9.93x10^{-26}$
	GO:BP	GO:0030036	actin cytoskeleton organization	$7.15x10^{-24}$
	GO:BP	GO:0007167	enzyme linked receptor protein signaling pathway	$8.91x10^{-25}$
	GO:BP	GO:0001944	vasculature development	$1.19x10^{-22}$
	GO:BP	GO:2000147	positive regulation of cell motility	$6.53x10^{-20}$
	GO:BP	GO:0001525	angiogenesis	$5.55x10^{-18}$
	GO:BP	GO:0097435	MAPK cascade	$3.29x10^{-22}$
	GO:MF	GO:0003779	actin binding	$2.23x10^{-9}$
	GO:MF	GO:0019900	kinase binding	$6.08x10^{-12}$
	GO:MF	GO:0050839	cell adhesion molecule binding	$3.89x10^{-8}$
	GO:MF	GO:0030695	GTPase regulator activity	$5.93x10^{-7}$
	GO:MF	GO:0005518	collagen binding	$9.12x10^{-4}$
	GO:MF	GO:0005543	phospholipid binding	$1.39x10^{-3}$

Supplementary Figure S5. Top Gene Ontology (GO) terms enriched in DEGs in cardiomyocytes and fibroblasts during myocardial infarction. The corrected P-values are shown on the colored boxes.

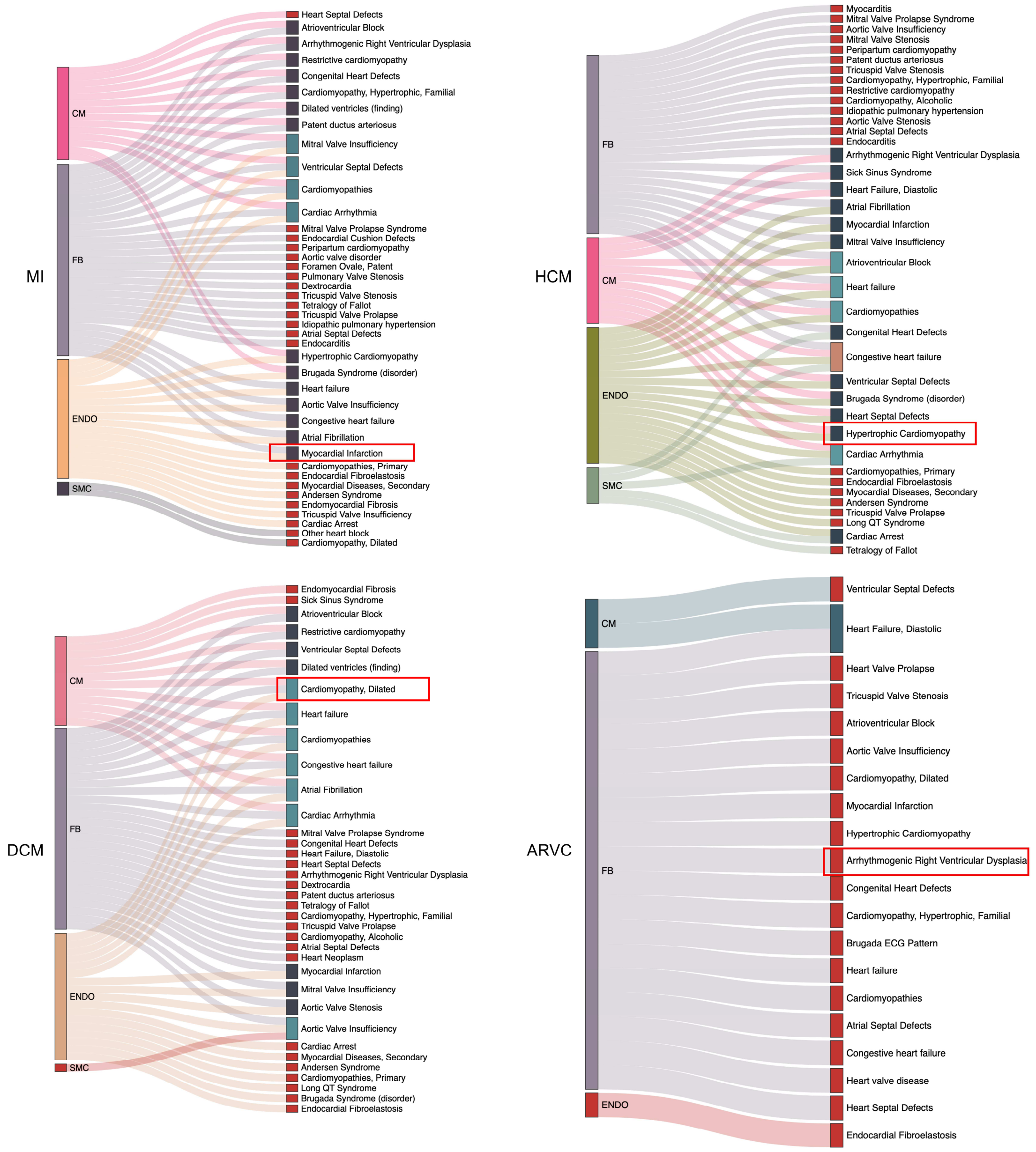

Supplementary Figure S6. Disease gene enrichment analysis revealing the cell type-specific cardiac disease gene associations.

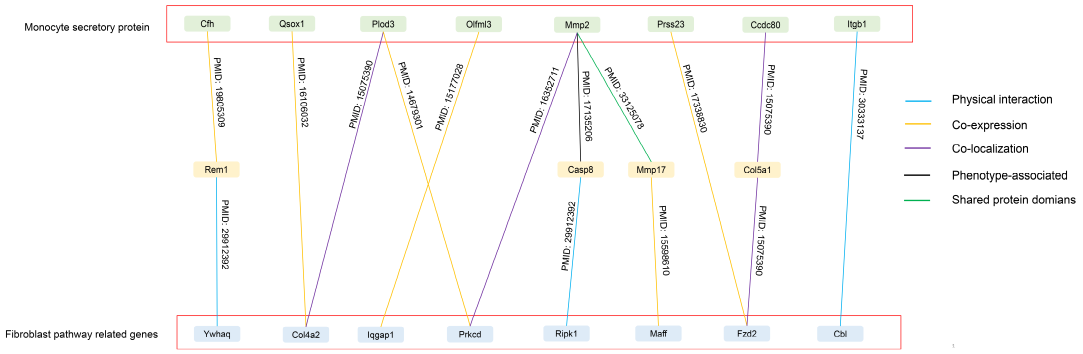

Supplementary Figure S7. Regulatory network between monocyte secretory proteins and fibroblast overrepresented pathway-related proteins. Different interaction categories are indicated by different edge colors. The PubMed ID (PMID) for literature evidence supporting the evidence is showing along the edge.
